# YY1’s role in the *Peg3* imprinted domain

**DOI:** 10.1038/s41598-017-06817-5

**Published:** 2017-07-25

**Authors:** Hongzhi He, An Ye, Bambarendage P. U. Perera, Joomyeong Kim

**Affiliations:** Department of Biological Sciences, Louisiana State University, Baton Rouge, LA 70803 USA

## Abstract

The ICR (Imprinting Control Region) of the *Peg3* (Paternally Expressed Gene 3) domain contains an unusual cluster of YY1 binding sites. In the current study, these YY1 binding sites were mutated to characterize the unknown roles in the mouse *Peg3* domain. According to the results, paternal and maternal transmission of the mutant allele did not cause any major effect on the survival of the pups. In the mutants, the maternal-specific DNA methylation on the ICR was properly established and maintained, causing no major effect on the imprinting of the domain. In contrast, the paternal transmission resulted in changes in the expression levels of several genes: down-regulation of *Peg3* and *Usp29* and up-regulation of *Zim1*. These changes were more pronounced during the neonatal stage than during the adult stage. In the case of *Peg3* and *Zim1*, the levels of the observed changes were also different between males and females, suggesting the different degrees of YY1 involvement between two sexes. Overall, the results indicated that YY1 is mainly involved in controlling the transcriptional levels, but not the DNA methylation, of the *Peg3* domain.

## Introduction

In mammals, a small subset of genes are functionally different between two alleles due to genomic imprinting, by which one allele is preferentially expressed in a parental-origin-specific manner^1^. These imprinted genes are usually clustered in specific regions of chromosomes, forming imprinted domains. Within a given domain, a small genomic region termed imprinting control region (ICR) is responsible for inheriting germ cell-driven DNA methylation as a gametic signal, and also for controlling the transcription of the entire domain as a *cis*-regulatory region^2^. Two DNA-binding proteins, CTCF (CCCTC-binding factor) and YY1 (Yin-yang 1), are known to bind to ICRs: CTCF to the ICR of the *H19*/*Igf2* (Insulin-like growth factor 2) domain and YY1 to the ICRs of the *Peg3* and *Gnas* (stimulatory G-protein alpha subunit) domains^3–6^. The DNA-binding sites of CTCF and YY1 within these ICRs are quite unusual. The number of these binding sites found within an ICR ranges from 4 to 10, which is much greater than the number in the other regulatory regions, such as enhancers and promoters. These binding sites in a given ICR are also all localized in a same orientation^7^. In the case of the ICR of *H19*/*Igf2*, the CTCF binding sites function as a methylation-sensitive insulator controlling the accessibility of the promoters of both *H19* and *Igf2* to the shared enhancers located in the 3′-side of *H19* ^8^. These CTCF binding sites are also required for maintaining the allele-specific DNA methylation of the ICR. Either loss of the CTCF sites or reduced levels of the CTCF protein during early embryogenesis resulted in DNA methylation on the active allele of the ICR, demonstrating critical roles played by CTCF for the functions of the ICR^9–12^.

The YY1 binding sites localized within the ICR of the *Peg3* domain have also been investigated through a series of *in vitro* and *in vivo* experiments. According to the results, reduced levels of the YY1 protein often resulted in changes in the DNA methylation levels of this ICR. Interestingly, the reduced levels of YY1 usually caused DNA hypomethylation in the ICR during oogenesis and also in cell lines and somatic tissues^13–15^. On the other hand, the YY1 binding sites have been shown to function as activators or repressors for the transcription of the *Peg3* domain depending upon the testing systems and also the functional contexts. For instance, a series of reporter assays using *in vitro* systems revealed that the transcriptional activity of the bidirectional promoter of *Peg3*/*Usp29* (Ubiquitin-specific protease 29) fluctuates, either up or down, depending upon the number of the YY1 binding sites involved in the activity^16^. In contrast, the reduced levels of YY1 protein usually resulted in the increased transcriptional levels of *Peg3*, suggesting that the YY1 binding sites might be repressors for the transcription of the *Peg3* domain^13–15, 17^. However, the interpretation of these results has not been straightforward since reducing the protein levels of YY1 might have impacted the *Peg3* locus in both direct and indirect ways, especially through the other loci that are also controlled through YY1.

To better understand the *in vivo* role of YY1 binding sites, thus, we have generated a mouse line carrying the mutated version of the 7 YY1 binding sites that are localized within the ICR of the *Peg3* domain. According to the results, these YY1 binding sites are not involved in the establishment and maintenance of the maternal-specific DNA methylation of the ICR. However, the YY1 binding sites appear to function as an activator for the transcription of the two adjacent genes, *Peg3* and *Usp29*. Interestingly, the transcriptional level changes observed from this mutant line were different between males and females, further suggesting the different degrees of YY1 involvement in the *Peg3* domain between the two sexes.

## Results

### Generation of an allele mutating the 7 YY1 binding sites within the Peg3-DMR

**Figure 1:**
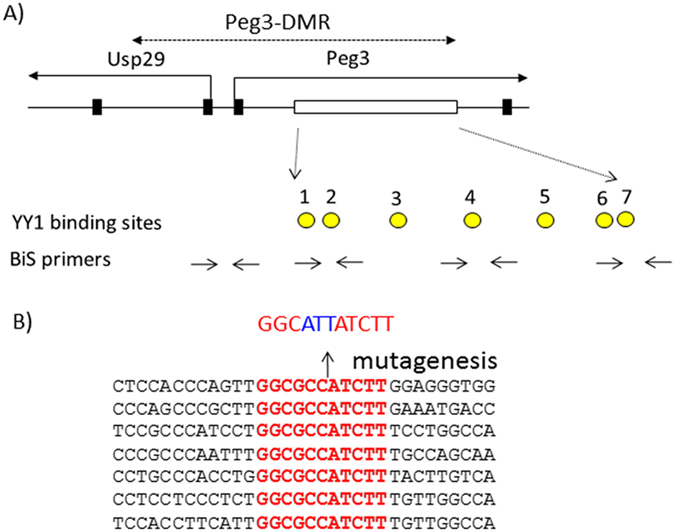
7 YY1 binding sites localized within the Peg3-DMR. (**A**) The 4-kb genomic region corresponding to the Peg3-DMR is indicated with a dotted line with arrows. The 1^st^ and 2^nd^ exons of both *Peg3* and *Usp29* are indicated with thick vertical lines, whereas the 2.5-kb YY1 binding region is indicated with an open box. The relative positions of the 7 YY1 binding sites are indicated with circles, and the positions of several sets of BiS (Bisulfite Sequencing) primers for DNA methylation analyses are indicated with arrows. (**B**) The sequence of the 7 YY1 binding sites are shown along with the flanking sequences. The sequences of the 7 YY1 binding sites with red are identical to each other. The three bases of each YY1 binding site were mutated from 5′-GCC-3′ to 5′-ATT-3′.

**Figure 2:**
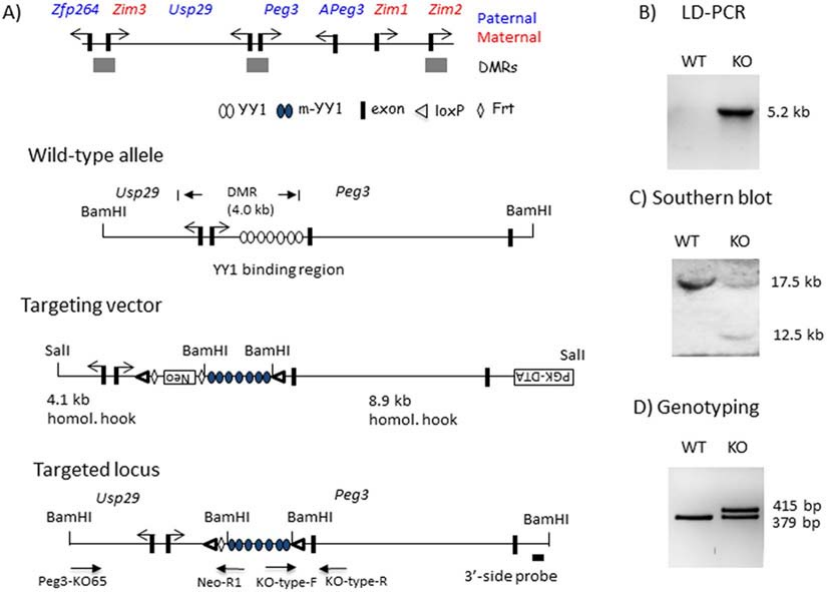
Peg3 domain and the targeting scheme. (**A**) Schematic representations of the *Peg3* domain (upper panel). Each imprinted gene is indicated with an arrow. The paternally and maternally expressed genes are indicated with blue and red, respectively. The three DMRs are indicated with gray boxes. Targeting scheme (lower panel). The 4.0-kb Peg3-DMR contains the first exons of *Peg3* and *Usp29* and the 2.5-kb YY1 binding region. The transcriptional direction of *Peg3* and *Usp29* is indicated with arrows, and exons are indicated with thick vertical lines. The region corresponding to the neomycin resistance gene (*NeoR*) along with the two flanking FRT sites within the targeting vector are indicated by an open box and diamonds, respectively. Arrows underneath ‘Targeted locus’ indicate primers with relative positions that were used for long-distance PCR and genotyping. (**B**) LD (Long Distance)-PCR. A set of primers, KO65 and NeoR1, successfully amplified the 5.2-kb genomic fragment from the genomic DNA of a heterozygote animal (KO), but not from that of a wild-type littermate (WT), confirming the proper targeting of the KO construct. (**C**) Southern blot analyses on the DNA isolated from ES cells that had been transfected with the targeting vector. The *BamH*I-digested DNA hybridized with the 3′side probe shows the wild-type allele (17.5 kb) and the targeted allele (12.5 kb). (**D**) PCR-based genotyping. The PCR scheme targeting the 2^nd^ loxP site with two primers (KO-type-F and -R) derived two DNA fragments representing the wild-type (379 bp) and KO (415 bp) alleles in the heterozygotes.

The ICR of the *Peg3* domain corresponds to the 4-kb genomic region encompassing the 1.5-kb bidirectional promoter of *Peg3*/*Usp29* and the 2.5-kb 1^st^ intron region of *Peg3* (Fig. 1). This ICR has been also termed the Peg3-DMR (Differentially Methylated Region) given its allele-specific DNA methylation pattern^5^. The 7 YY1 binding sites within the 2.5-kb intron region were previously mutated for a series of *in vitro* reporter assays^16^. The three bases of each binding site was changed from 5′-GCC-3′ to 5′-ATT-3′, which is part of the core binding motif for YY1^18^. The 2.5-kb region containing the mutant version of 7 YY1 binding sites has been used to construct a targeting vector for mouse knockout (KO) experiments (Fig. 2A). The final targeting vector was transfected into the ES cells of 129/SvJ. Transfected ES cells were subsequently screened with long-distance PCR and southern blotting, identifying 20 targeted clones out of 300 ES cells (Fig. 2B,C). Two independent clones with the proper targeting were injected into the blastocysts of C57BL/6 J, subsequently generating 10 chimeras. Among these chimeras, two were able to generate F1 pups with the germline transmission of the targeted allele. The F1 mice were further bred with a Flippase line to remove the *NeoR* (Neomycin Resistance) cassette, which is located between the 1.5-kb bidirectional promoter and the 2.5-kb intron regions. The 3-bp-mutation, 5′-GCC-3′ to 5′-ATT-3′, in each of the 7 YY1 binding sites was confirmed again through sequencing of the genomic DNA isolated from the F1 mice (data not shown). Besides the mutated 7 YY1 binding sites, the final mutant allele also contains two loxP sites flanking the 2.5-kb intron region. Thus, a set of primers amplifying the 379-bp region surrounding the 2^nd^ loxP site was used to genotype all the progeny from the current study (Fig. 2D). Overall, we have successfully generated a mouse allele containing the mutant version of the 7 YY1 binding sites for the ICR of the *Peg3* domain.

### Phenotypic effects of the mutant allele at the organism level

The phenotypic effects of the mutant allele were analyzed at the organism level using a series of breeding experiments (Supplemental material 1). Male and female heterozygotes were bred individually with their female and male wild-type littermates. The subsequent pups with the paternal and maternal transmission of the mutant allele were analyzed in terms of their sex, genotype and body weight. The results are summarized as follows. First, the average litter size of the pups with the paternal transmission of the mutant allele was smaller than the average litter size of the 129/B6 mice (7.76 vs 10), yet the ratio between the wild-type and heterozygote was close to the mendelian ratio (WT:KO = 28:26), indicating no major embryonic lethality associated with the mutant allele. This is also the case for the maternal transmission of the mutant allele. The average litter size was 7.6, and the ratio between the wild-type to heterozygote was also close to the mendelian ratio (WT:KO = 21:25). Second, the weight profiles of one-day-old neonates also showed no major differences between WT and KO in both the paternal and maternal transmission (Supplemental material 1). Interestingly, the male heterozygotes from the paternal transmission were slightly heavier than those from the wild-type littermates (104% with 12% S.D. versus 100% with 7% S.D., respectively), but this observation was not statistically significant (Student t-test, *p* = 0.075). It is also prudent to note that the mutant strain used for this study is the mixed background 129/B6. Thus, the outcome needs to be interpreted with caution. Overall, the mutation on the 7 YY1 binding sites did not cause any major effects on the survival and growth rates of the mice.

### Mutational effects on DNA methylation levels

**Figure 3:**
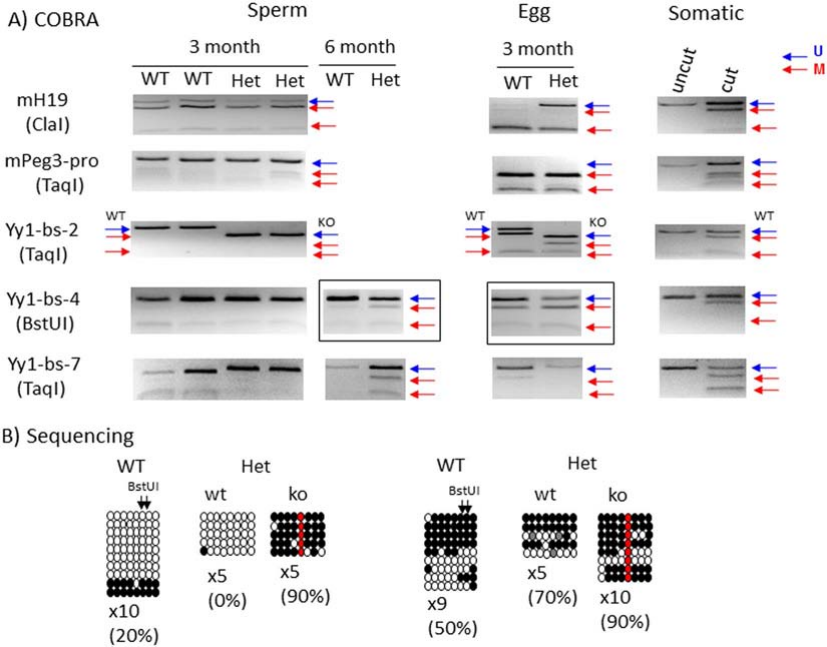
Mutational effects on DNA methylation levels. (**A**) A series of DNA methylation analyses were performed using the DNA isolated from sperm and mature oocytes. The DNA isolated from germ cells was treated with the bisulfite conversion protocol, and subsequently used for the amplification of each target region. The amplified PCR product was analyzed with COBRA. The restriction enzyme used for each set of PCR products is shown underneath of the name of each target. The unmethylation and methylation based on the digestion pattern by a given restriction enzyme are also indicated by blue and red arrows, respectively. This series of COBRA analyses also included somatic DNA as a control set of DNA, which can monitor the proper execution of the experiments. The DNA methylation levels of the H19-ICR were used as an internal control monitoring the purity of sperm and egg. The H19-ICR is mostly methylated in sperm whereas unmethylated in egg. The PCR products from sperm and eggs by the Yy1-bs-4 set, which were marked with boxes, were further analyzed by individual sequencing. These results are shown in (**B**). The sequencing results from these products were summarized in the following manner. Each row represents one sequence read while each circle within this row indicates one CpG dinucleotide. The open and close circles indicate the unmethylated and methylated CpG sites, respectively. The red circles indicate the CpG site within #4 YY1 binding site, which has been mutated to a non-CpG dinucleotide. The gray circles indicate the CpG sites with ambiguous base-calling. This mutation was used to separate the sequence reads into two groups, the mutant (ko) and wild-type (wt) alleles. The ‘X’ in 5X or 10X indicates the number of clones that were sequenced after the cloning of each PCR product. The percentage values indicate the methylation levels that have been measured by sequencing. In this set of DNA methylation analyses, we were not able to amplify one PCR product, one by mH19 primer set from the WT egg. Also, the sizes of some of the final products are different between WT and KO due to the inclusion of a loxP site between two primers, the products by the Yy1-bs-2 and Yy1-bs-7 primer sets. In the case of Yy1-bs-2, a separate primer set targeting the KO allele was used for the Het samples of both sperm and eggs, thus generating a different-size product. The original gel images for the gel pictures are available as Supplemental material 6.

**Figure 4:**
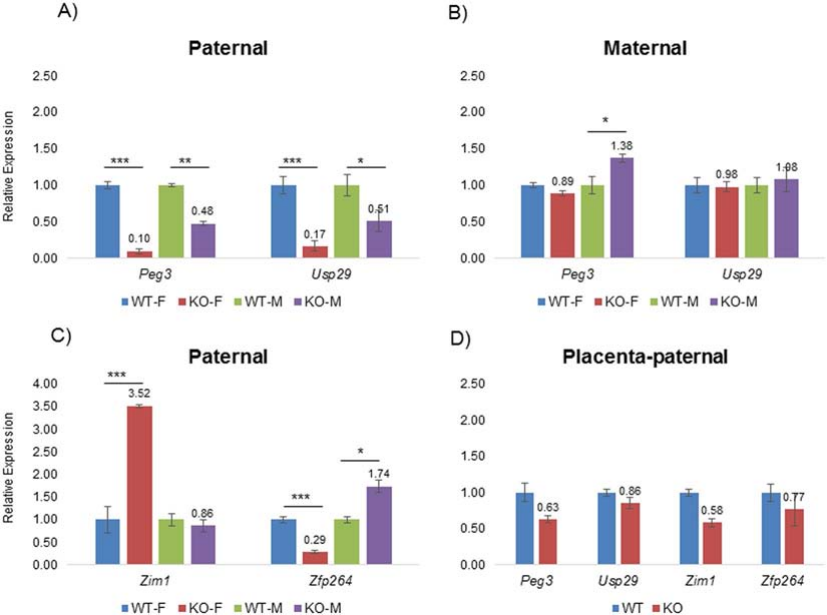
Mutational effects on transcriptional levels. A series of qRT-PCR were performed to test potential effects of the mutation on the transcriptional levels of the imprinted genes within the *Peg3* domain. This series of analyses used the total RNA isolated from the brain of the one-day-old pups carrying the mutant allele paternally (**A**,**C**) and maternally (**B**). The expression levels of each gene were first normalized with an internal control (β-actin), and the normalized values were further compared between the wild-types and the heterozygotes. The relative levels are presented in a graph with standard errors (S.E.). A similar series of expression analyses were also conducted using a set of 14.5-dpc placenta (**D**). Statistical significance by Student’s t-test was indicated in the following manner: * < 0.05, ** < 0.01, *** < 0.001. The series of analyses were repeated with two independent sets of biological replicates.

Potential effects of the mutation on the DNA methylation levels of the Peg3-DMR were analyzed in the following manner. First, we isolated the DNA from the sperm that had been purified from three sets of adult males. Each set has two heterozygotes and wild-type littermates. The first two sets were from 3-month-old mice, whereas the third set from 6-month-old mice. The isolated DNA were first treated with the bisulfite conversion protocol, and later used as templates for PCR to amplify several target regions within the Peg3-DMR (Figs 1 and 3). We targeted three YY1 binding sites (Yy1-bs-2, -4, -7) as well as the bidirectional promoter region of *Peg3*/*Usp29* (mPeg3-pro) (Fig. 3). We have also included the ICR of the *H19*/*Igf2* domain (mH19) as an internal control to monitor the purity of the isolated sperm (Fig. 3). The amplified PCR product from each locus was analyzed with COBRA (COmbined Bisulfite and Restriction Analysis^19^), and later by individual sequencing (Fig. 3B). The sperm from the two sets of 3-month-old mice did not show any major difference between the wild-type and heterozygotes, showing no methylation in the Peg3-DMR. However, the sperm from the 6-month-old heterozygote mice showed about 50% methylation at two regions, Yy1-bs-4 and -7, but not from the sperm of the wild-type littermates. The results from individual sequencing also revealed that DNA methylation was detected exclusively from the mutant allele, but not from the wild-type allele, of the heterozygotes (Fig. 4B). This suggests that the intact YY1 binding sites may be required for protecting DNA methylation during the spermatogenesis of the older mice.

Second, we also repeated a similar series of DNA methylation analyses using the mature eggs that had been isolated from three sets of females. Each set was comprised of three wild-type and heterozygote females of the 3-month-old age. In a given set, we successfully obtained 60–80 eggs for each of the two groups, wild-type and heterozygote, which were subsequently used for DNA methylation analyses (Fig. 3). The results revealed no major difference between the wild-type and heterozygote, both showing proper DNA methylation on the Peg3-DMR. Individual sequencing further indicated that the two alleles within the heterozygotes also showed similar levels of DNA methylation between the wild-type and mutant alleles (Fig. 3B). Thus, this further confirmed that the YY1 binding sites may not be required for the establishment of oocycte-specific DNA methylation on the Peg3-DMR. Third, we also performed a series of COBRA using the DNA isolated from the individual tissues of the adult mice (Supplemental material 2). However, we did not observe any difference in the DNA methylation levels of the Peg3-DMR between the wild-type and heterozygote, confirming again that the YY1 binding sites may be dispensable for the maintenance of the allele-specific DNA methylation of the Peg3-DMR. This has been further confirmed through a series of imprinting tests using the hybrids that had been derived from the crossing of the male heterozygote of the 129/B6 background with the female breeders of the PWD/PhJ strain (Supplemental material 3). No change was observed in the imprinting status of the genes in the *Peg3* domain. Taken together, this series of analyses concluded that the 7 YY1 binding sites are not required for the establishment of the allele-specific DNA methylation of the Peg3-DMR during oogenesis, but that some of these YY1 binding sites may be required for protecting DNA methylation during the spermatogenesis.

### Mutational effects on transcriptional levels

We also analyzed the potential effects of the mutation on the transcriptional levels of the genes within the *Peg3* domain. Total RNA was isolated from the brains of the two sets of one-day-old pups for cDNA synthesis (Fig. 4). The first set includes the wild-type and heterozygote of both sexes with the paternal transmission of the mutant allele (Fig. 4A,C), whereas the second set includes the same combination of pups but with the maternal transmission of the mutant allele (Fig. 4B). These two sets of cDNA were used for performing qRT-PCR with several sets of primers targeting the imprinted genes within the *Peg3* domain, including *Peg3*, *Usp29*, *Zim1* (Zinc finger protein 1, imprinted) and *Zfp264* (Zinc finger protein 264). The other two remaining genes, *Zim2* (Zinc finger protein 2, imprinted) and *Zim3* (Zinc finger protein 3, imprinted), were not included for this analysis due to the very low expression levels in neonatal brains. This series of expression analyses derived the following results. First, the paternal transmission of the mutant allele resulted in the down-regulation of both *Peg3* and *Usp29*, suggesting that the YY1 binding sites may function as a transcriptional activator (Fig. 4A). Interestingly, the levels of the observed down-regulation were not similar between two sexes: 10% (female) vs 48% (male) for *Peg3* whereas 17% (female) vs 51% (male) for *Usp29*. The mutant allele also caused a similar sex-biased outcome in the expression levels of the two adjacent genes, *Zim1* and *Zfp264*: 350% (female) and 89% (male) for *Zim1* whereas 29% (female) and 174% (male) for *Zfp264* (Fig. 4C). Second, the maternal transmission of the mutant allele did not cause any major effects on the expression levels of the surrounding genes (Fig. 4B), which is consistent with the fact that the maternal allele of the Peg3-DMR harboring the mutated YY1 binding sites are inactive due to DNA methylation. Third, we also analyzed the mutational effects using the total RNA isolated from placenta (Fig. 4D) and also adult tissues, including the brains and hearts of 2-month-old male and female mice (Supplemental material 4). According to the results, the mutation resulted in the down-regulation of all the genes tested in placenta: *Peg3* (63%), *Usp29* (86%), *Zim1* (58%) and *Zfp264* (77%). Among these changes, the expression level changes of *Peg3* and *Zim1* were statistically significant. In the case for adult tissues, both *Peg3* and *Usp29* were down-regulated in the brain and heart of adult mice (Supplemental material 4). Overall, this series of expression analyses concluded that the mutation on YY1 binding sites resulted in the overall down-regulation of *Peg3* and *Usp29* in the tissues of neonatal and adult stages. Interestingly, the levels of the observed down-regulation of *Peg3* and *Usp29* differ between males and females in neonatal brains, suggesting that the functional involvement of YY1 in the *Peg3* domain might be different between the two sexes.

### Mutational effects on chromatin structure

**Figure 5:**
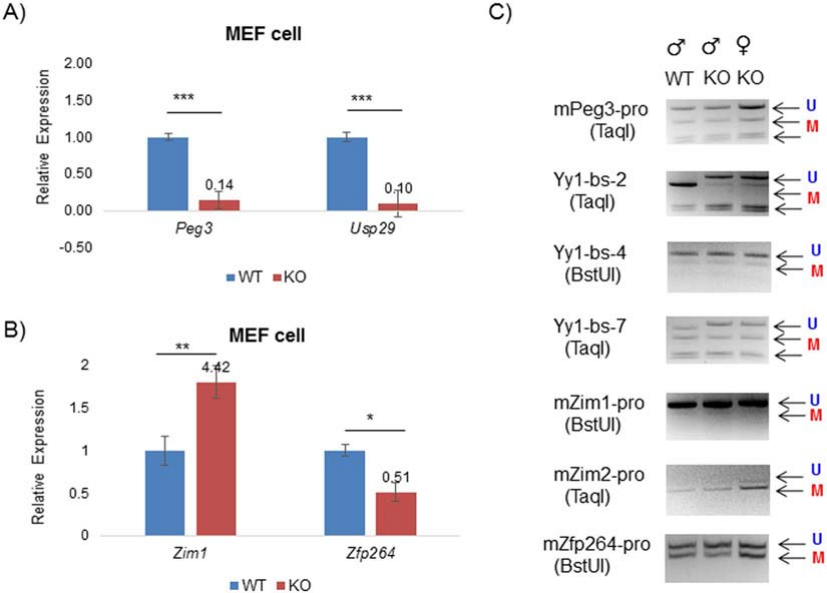
Mutational effects in MEF cells. (**A**,**B**) A set of male MEF cells (WT and KO) were used to test potential effects of the mutation on the expression levels of the imprinted genes. The expression levels of each gene were first normalized with an internal control (β-actin), and the normalized values were further compared between the wild-types and the heterozygotes. The relative levels are presented in a graph with standard errors (S.E.). Statistical significance by Student’s t-test was also indicated in the following manner: * < 0.05, ** < 0.01, *** < 0.001. (**C**) A set of DNA isolated from male and female MEF cells were used for surveying the DNA methylation levels of the imprinted genes with COBRA. The restriction enzyme used for each set of PCR products is shown underneath of the name of each target. The unmethylation and methylation status based on the digestion pattern by a given restriction enzyme is also indicated by blue U and red M letters with arrows, respectively. The original gel images for the gel pictures are available as Supplemental material 6.

The mutational effects were further analyzed using MEFs (Mouse Embryonic Fibroblasts) derived from a set of 13.5-d.p.c. embryos that had been prepared through timed mating between male heterozygotes and female breeders (Fig. 5). First, the total RNA isolated from the male and female sets of MEFs, WT and KO, were used for measuring the expression levels of the genes within the *Peg3* domain. The result from the male set is shown as a representative set since the changes observed from two sexes were similar (Fig. 5A,B). The outcome is overall similar to those from neonatal brains: the dramatic down-regulation of *Peg3* (14%) and *Usp29* (10%) and the concurrent up-regulation of *Zim1* (442%). The mutation also resulted in the less dramatic down-regulation of *Zfp264* (51%). The observed down-regulation of *Peg3* and *Usp29* was consistent with the patterns from neonatal and adult tissues. The observed changes in the expression levels were also analyzed in terms of their DNA methylation levels (Fig. 5C). The results revealed no major change in the DNA methylation levels of the imprinted genes in both female and male MEFs, suggesting that any changes in the expression levels of the imprinted genes are likely caused by some unknown factors other than DNA methylation.

**Figure 6:**
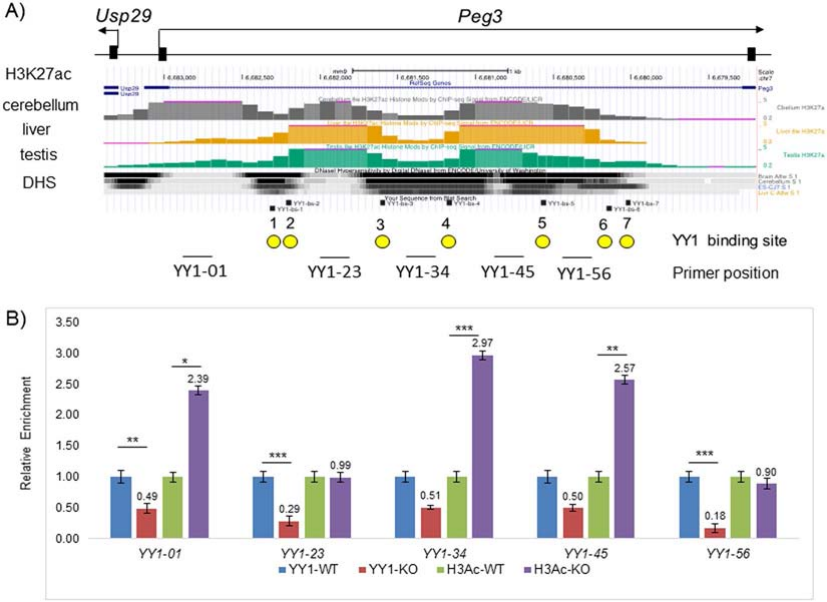
Mutational effects on the levels of YY1 binding and H3K27ac. (**A**) The diagram summarizes the profiles of the H3K27ac modification and the DNaseI-hypersensitivity (DHS) associated with the Peg3-DMR. The grey, orange, green peaks represent the H3K27ac profiles from cerebellum, liver, and testis, respectively. The three layers of thick lines underneath the graphs represent the profiles of DNaseI-hypersensitivity from cerebellum, liver and testis with the darkest areas being the most sensitive regions. The relative positions of primer sets used for this study are shown along with the genomic structure of the Peg3-DMR. (**B**) A series of ChIP experiments were performed using the chromatin derived from a set of WT and KO MEF cells. The antibodies against YY1 and H3K27ac were individually used to test the binding of YY1 and also to compare the levels of H3K27ac between WT and KO. The enrichment levels were first normalized with those of the input DNA, and later these normalized values were compared between WT and KO. These relative values are summarized and presented in bar graphs with standard errors (S.E.). Statistical significance by Student’s t-test was also indicated in the following manner: * < 0.05, ** < 0.01, *** < 0.001.

We further followed up the potential effect of the mutation on the chromatin structure of the Peg3-DMR in the following manner. The histone modification profiles of the Peg3-DMR are already available as part of the Epigenome project (Fig. 6A). According to the available data, the 2.5-kb YY1 binding region is represented by two H3K27ac peaks, and these two peaks are also surrounded by several DNaseI-hypersensitive sites, which are marked as dark areas. Interestingly, YY1 binding sites tend to be located in the border regions between the H3K27ac peaks and DNaseI-hypersensitive sites, suggesting that YY1 may be involved in the formation and/or maintenance of the chromatin environment of the Peg3-DMR. To test this possibility, we performed two sets of ChIP (Chromatin ImmunoPrecipitation) experiments. First, we tested whether the mutation indeed abrogates the binding of YY1 to the Peg3-DMR. The chromatin prepared from a set of WT and KO MEF cells were immunoprecipitated with anti-YY1 polyclonal antibody, and the precipitated DNA was analyzed with qPCR analyses (Fig. 6B). As expected, the levels of the enrichment were much lower in the KO MEF than the levels in the WT MEF at all of the YY1 binding sites, confirming that the mutation indeed abrogated the binding of YY1 to the Peg3-DMR. Second, a similar series of ChIP experiments were also performed with anti-H3K27ac antibody (Fig. 6B). According to the results, the three regions showed high levels of the H3K27ac modification in the KO MEF than in the WT MEF, including YY1-01, YY1-34 and YY1-45. In contrast, two regions showed no difference between the two pools of MEFs, YY1-23 and YY1-56. The higher levels of H3K27ac modification observed from the KO MEF was unexpected since the mutation causing the down-regulation of both *Peg3* and *Usp29* should, intuitively, correlate well with the loss of the activation signals of histone modifications, such as H3K27ac. Although unexpected, the results are overall consistent with the initial prediction that YY1 may be involved in the formation of the proper chromatin environment of the Peg3-DMR. Taken together, this series of analyses using MEFs concluded that the mutation resulted in the down-regulation of *Peg3* and *Usp29* and also caused the changes in the histone modification levels associated with the Peg3-DMR.

## Discussion

In the current study, we have mutated the 7 YY1 binding sites localized within the Peg3-DMR. According to the results, the mutation resulted in the down-regulation of the two adjacent genes, *Peg3* and *Usp29*, in the majority of the tissues harvested at different developmental stages, suggesting YY1 as an activator for the transcription of the two genes. In contrast, the mutation did not cause any effect on the allele-specific DNA methylation of the Peg3-DMR, confirming that YY1 is not involved in the DNA methylation of the *Peg3* domain. Overall, the current study concluded that YY1 is mainly involved in controlling the transcriptional levels of the *Peg3* domain.

The unusual cluster of multiple YY1 binding sites within the Peg3-DMR has been a very unique feature that has been conserved during mammalian evolution^7^. Similar clusters of YY1 and CTCF binding sites are also present within the ICRs of the other imprinted domains, including the *Gnas* and *H19*/*Igf2* domains^7^. Thus, these clusters of YY1 and CTCF binding sites have been thought to be involved in some unknown functions that are unique to genomic imprinting. One such function may be related to the establishment and maintenance of allele-specific DNA methylation. This prediction has been further supported by CTCF’s roles in protecting DNA methylation from the active maternal allele of the ICR of the *H19*/*Igf2* domain^9, 11^. A series of similar observations have been also made from the YY1 binding sites in the *Peg3* and *Gnas* domains, revealing that reducing the protein levels of YY1 caused either DNA hypomethylation or hypermethylation on the ICRs of the *Peg3* and *Gnas* domains^13–15^. Despite all these previous observations, the current study clearly demonstrated that the YY1 binding sites within the Peg3-DMR are not directly involved in the establishment and maintenance of the allele-specific DNA methylation on the Peg3-DMR (Fig. 3). This further corrects the previous interpretation of the results that were derived from a series of YY1 knockdown experiments^13–15^. In those experiments, the changes observed in the DNA methylation levels of the Peg3-DMR may have been an indirect outcome through some unknown loci that are controlled by YY1. On a separate note, however, a subset of the male heterozygotes was shown to produce sperm with DNA hypermethylation on the Peg3-DMR (Fig. 3). Interestingly, a similar result was also observed from the previous experiments, displaying DNA hypermethylation on the Peg3-DMR in the sperm that had underwent conditional knockdown of YY1 during spermatogenesis^15^. Thus, it is still possible that some of the YY1 binding sites may be involved in protecting the encroachment of DNA methylation from the adjacent regions to the Peg3-DMR. This is reminiscent of the case of the CTCF sites within the ICR of the *H19*/*Igf2* domain^9, 11^. Overall, the current study along with the previous studies suggest that one of the shared functions between YY1 and CTCF in genomic imprinting may be protecting the active allele of ICRs from DNA methylation.

The results from the tissues of neonatal and adult stages clearly demonstrated that both *Peg3* and *Usp29* are down-regulated in the mutant mice, suggesting that the YY1 binding sites function as an activator for the transcription of *Peg3* and *Usp29* (Figs 4 and 5). This down-regulation was also accompanied with the changes in the transcription levels of the adjacent genes within the *Peg3* domain, including *Zim1* and *Zfp264* (Fig. 4). *Zim1* was up-regulated in the mutants, whereas *Zfp264* was either up and down-regulated depending upon the individual tissues tested. These changes observed from *Zim1* and *Zfp264* are consistent with the pattern observed from the other ICRs, further supporting the fact that the Peg3-DMR with the 7 YY1 binding sites is responsible for controlling the transcription of the entire *Peg3* domain. On the other hand, the current study also provides one unique observation, which has not been observed from the other ICRs. The levels of the down-regulation of *Peg3* and *Usp29* were different between males and females (Fig. 4A). The mutational effects on females observed in neonatal brains were more severe than those on males, which might be also responsible for the different responses from the two neighboring genes, *Zim1* and *Zfp264*. It has also been known that the *Peg3* locus is sex-specific in many ways. For instance, the expression levels of *Peg3* itself were different between two sexes, showing higher in males than in females^20^. Also, the mutations and environmental stresses affecting the *Peg3* locus have been shown to affect more severely the survival of males than females^21, 22^. The current study further suggests that YY1 might be the main culprit causing these sex-biased effects, which will be interesting to pursue in the near future. Overall, the current study concluded that the main role of the YY1 binding sites within the Peg3-DMR is controlling the transcriptional levels of *Peg3* and *Usp29*.

Despite numerous studies, it is still enigmatic why the Peg3-DMR and the other ICRs have maintained a cluster of multiple YY1 or CTCF binding sites during mammalian evolution. Two scenarios have been previously proposed for this unusual multiplicity^7^. The first scenario is that multiple DNA-binding sites for YY1 or CTCF may be needed for the maintenance of the epigenetic modifications for relatively large-size regulatory regions, ICRs, which range from 2 to 4 kb in length. The results from the current study support this possibility since the mutation on YY1 binding sites rendered the encroachment of DNA methylation from the adjacent region to the Peg3-DMR during spermatogenesis (Fig. 3). Also, the available histone modification profiles indicate that the positions of all of the YY1 binding sites within the Peg3-DMR coincide very well with the border regions between histone modification peaks and DNaseI-hypersensitive regions (Fig. 6A). These lines of evidence clearly support an idea that the multiple YY1 binding sites are involved in the formation of the chromatin environment of the Peg3-DMR. The second scenario is that the multiple YY1-binding sites within the Peg3-DMR might be designed to secure a sufficient level of the YY1 protein around this ICR. Yet, this protein level of YY1 around the Peg3-DMR might be a very critical factor deciding the body size of animal pups. This is likely since the gene dosage of *Peg3* is known to correlate very well with the body size of animal pups^21, 23^. In this case, the increased levels of the YY1 protein through additional YY1 binding sites might boost the body size and thus the survival chance of animals. On the other hand, the unusually high levels of the YY1 protein could cause the unsustainable size of the fetus, which might jeopardize the health of the pregnant females. To counter this hazardous outcome, the maternal genome might need to reduce the number of YY1 binding sites through DNA methylation-mediated substitutions from CpG to TpG or CpA within the YY1-binding site (CGCCATnTT), which has been shown to reduce dramatically the binding affinity of YY1 to DNA^24^. Consistent with this, the Peg3-DMRs of many mammals, including human and mouse, have several imperfect YY1 binding sites with one or two base differences from the consensus sequence. This decay process of CpG sites during evolution is particularly feasible since the Peg3-DMR becomes methylated as part of genomic imprinting during oogenesis. If this scenario turns out to be the case, the multiple YY1 binding sites detected in a given species might be simply reflecting the outcome of this evolutionary tug-of-war between two parental genomes with opposite needs^25, 26^. Further investigation of this possibility would be of great interest, but may require different experimental approaches involving phylogenetic and comparative genomics. Overall, the observations from the current study are consistent with both scenarios, which are non-exclusive. Therefore, we conclude that the multiple YY1 binding sites within the Peg3-DMR may have been selected for both functional needs, maintaining the chromatin environment and securing a sufficient level of the YY1 protein around the Peg3-DMR.

## Materials and Methods

### Ethics Statement

All the experiments related to mice were performed in accordance with National Institutes of Health guidelines for care and use of animals, and also approved by the Louisiana State University Institutional Animal Care and Use Committee (IACUC), protocol #16-060.

### Generation of a knockout allele

The targeting vector for the knockout experiment was constructed with the RED/ET recombination technique (Gene Bridges; ref. 27). A mouse BAC (bacterial artificial chromosome) clone, RP23-178C5 (Invitrogen), was used as a source for isolating the 15.4-kb genomic fragment surrounding the Peg3-DMR (nucleotide positions 6,718,442-6,733,840 in the mouse chromosome 7 of mm10). The 15.4-kb fragment was isolated through homologous recombination using two hooks, which were part of the two following oligonucleotides: GB primer 1 (5′-GCAAACGCCGTGTTATCAAACACCTTCATCTCAGACCACGGTCTGTGCTG-*GTCGAC-*ACAGCTTGTCTGTAAGCGGATG-3′) and GB primer 2 (5′-CAAAACAGACAACTGTGAAAAACTCACCACTCCGTTGGAGAGTTTCAAGA- *GCGGCCGC-*GCTCTCCTGAGTAGGACAAATCCG-3′). The 50 nucleotide-long sequences at the 5′-ends of both primers were the homology hooks while the sequences at the 3′-end of both primers were included for the amplification of a minimal cassette for the RED/ET recombination system (Gene Bridges, Cat. No. K002). Two restriction enzyme sites, *Sal*I and *Not*I (italicized regions), were also included as part of the sequences to be used for the linearization of the final vector and the cloning of the negative selection marker DTA (Diphtheria toxin A), respectively. PCR amplification of the minimal cassette with these two primers generated the linearized minimal cassette with the two homology hooks at its 5′- and 3′-ends. The E. coli strain carrying the BAC RP23-178C5 was transformed with the expression plasmid pRedET and the linearized minimal cassette containing the two homology hooks. Several colonies containing the circularized 17.8-kb vector (15.4-kb target fragment plus the 2.4-kb minimal cassette) were obtained through the ampicillin selection. The integrity of the isolated 15.4-kb genomic fragment was further confirmed through a series of restriction enzyme digestions. One round of additional pRedET-based recombination was performed to replace the 2.5-kb YY1 binding region with the neomycin resistance gene (*NeoR*) that is flanked by FRT sites. This replacement process has also incorporated one loxP site at the 5′-end and one *BamH*I site at the 3′-end (Fig. 2A). The subsequent vector was further modified by inserting the mutant version of the YY1 binding region into the *BamH*I site. The mutant version of the YY1-binding region, which was previously generated^16^, was amplified with the two primers containing *BamH*I site for subcloning. During this subcloning step, another loxP site has been inserted at the 3′-side, thus this newly inserted loxP site can be paired with the other loxP site that had been inserted earlier at the 5′-side of the 2.5-kb YY1 binding region. Finally, we subcloned the expression cassette DTA into the *Not*I site as a negative selection marker. The final 21.4-kb KO vector was linearized with *Sal*I digestion, and subsequently used for transfection into the AB2.2 ES cell line of the 129/SvJ origin (http://www.bcm.edu/dtmc/, Darwin Transgenic Mouse Core facility of Baylor College of Medicine). Transfected ES cells were first screened with a long-distance PCR scheme that can confirm the proper recombination of the 5′-side genomic fragment with the following primer set: Peg3-KO-65 (5′-TTCCTAAAGGCAAGTAGGACCT-3′) and Neo-R1 (5′-GATTCGCAGCGCATCGCCTTCT-3′). Later, a subset of the potential targeted clones identified through LD-PCR were further analyzed with southern blotting to confirm the proper recombination of the 3′-side genomic fragment. For this southern blotting, the DNA isolated from ES cells were digested with *BamH*I, and probed with the 495-bp fragment that had been amplified by PCR with the following primer set: Peg3-KO-63 (5′-ACCTTCCACTAGATTTCACCTCCT-3′) and Peg3-KO-64 (5′-CACTGCCAAAAGCATGAGATGGTC-3′). Two targeted ES cell was microinjected into the blastocysts of e3.5-embryos of the C57BL/6 J (B6) mouse, generating ten chimeras with varying degrees of coat color contribution. Four of these chimeras were bred with 8 B6 females, finally deriving F1 mice with germline transmission of the targeted allele.

### Mouse breeding

The male and female heterozygotes carrying the mutant allele were bred individually with female and male wild-type littermates. One-day-old pups derived from these breeding experiments were analyzed in terms of sex, genotype and body weight. Statistical significance of potential difference of litter size and average weight between two breeding experiments was tested Χ^2^-test. All the mice were housed at the DLAM (Division of Lab Animal Medicine) of LSU on a regular 12-12 dark-light cycle under a constant temperature 70°F and 50% humidity. All animals were given ad libitum access to water and Rodent Diet 5001. The nursing females were with Mouse Diet 5015. The mice were euthanized by CO2 asphixation in accordance with the rules and regulations set forth by the IACUC. For genotyping, genomic DNA was isolated from either clipped ears by incubating the tissues overnight at 55 °C in the lysis buffer (0.1 M Tris-Cl, pH 8.8, 5 mM EDTA, pH 8.0, 0.2% SDS, 0.2 M NaCl, 20 μg/ml Proteinase K). The isolated DNA was subsequently genotyped using the following primer set: KO-type-F (5′-ATGACAAGTGGGCTTGCTGCAG-3′) and KO-type-R (5′-GGATGTAAGATGGAGGCACTGT-3′). The sexes of the pups were determined through PCR using the following primer set: mSry-F (5′-GTCCCGTGGTGAGAGGCACAAG-3′) and mSry-R (5′-GCAGCTCTACTCCAGTCTTGCC-3′).

### Expression analyses and imprinting test

Total RNA was isolated from the tissues of one-day-old heads and adult mice using a commercial kit (Trizol, Invitrogen). The total RNA was then reverse-transcribed using the M-MuLV kit (Invitrogen), and the subsequent cDNA was used as a template for quantitative real-time PCR. This analysis was performed with the iQ SYBR green supermix (Bio-Rad) using the ViiA™ 7 Real-Time PCR System (Life Technologies). All qRT-PCR reactions were carried out for 40 cycles under standard PCR conditions. The analyses of the results derived from qRT-PCR were described previously^13^. Statistical significance of potential difference of expression levels of a given gene between two samples was tested Student’s t-test. The information regarding individual primer sequences and PCR conditions has been published in the previous study^21^. For imprinting test, the heterozygotes of the 129/B6 background were bred with the PWD/PhJ strain (Jackson Lab, Stock No. 004660). The F1 hybrid of this crossing was used for isolating total RNA. The polymorphisms and restriction enzymes used for each gene’s imprinting test are also available through the previous study^21^.

### DNA methylation analysis

DNA methylation levels of each target region were analyzed using genomic DNA isolated from two germ cells, sperm and oocyte, and also the tissues of the neonates and adult mice. Detailed protocols for isolating sperm and oocyte were described previously^15^. Briefly, sperm was isolated from the epididymus of 3 and 6-moth-old mice using the ‘swim-up’ method^28^. Mature oocytes were isolated from 3-month-old females after superovulation with PMSG and hCG treatment^29, 30^. The isolated DNA was treated with the bisulfite conversion reaction according to the manufacturer’s protocol (EZ DNA methylation kit, Zymo Research). The converted DNA was used as a template for the PCR reaction using specific primers that were designed for amplifying each target region. The majority of PCR amplifications were carried out using a standard protocol with 40 cycles, whereas the PCR amplification from eggs was conducted using a nested scheme with the initial step with 40 cycles followed by the second step with 30 cycles. Each PCR product was further analyzed using the following two approaches: 1) the restriction enzyme digestion-based COBRA^19^ and 2) subcloning and sequencing. For the COBRA analysis, each PCR product was digested with a series of restriction enzymes. The PCR product was also individually subcloned into the pGEM T-Easy vector (Promega), and 10 to 20 clones were subsequently sequenced to survey its DNA methylation levels at each locus. The detailed information regarding oligonucleotide sequences, sequence polymorphisms, and COBRA is also available (Supplemental material 5).

### Derivation of MEF (Mouse Embryonic Fibroblast) cells

Two litters of 13.5-dpc embryos of the 129/B6 background were harvested through timed mating of the male KO heterozygotes with the female wild-type littermates. The head portion and the red tissues were removed from the embryos, and the remaining portions were minced with razor blades. These minced tissues were transferred to a 15 ml conical tubes containing 1 ml trypsin (Invitrogen, Cat. No. 25300062). After 5 min incubation at 37 °C, the cells were harvested with centrifugation, and later resuspended in 15 ml media (Life technologies, Cat. No.10566024). Finally, the resuspended cells were plated onto a T-75 flask. The MEF from each embryo was first genotyped using the following primer set: Peg3-KO-63 (5′-ACCTTCCACTAGATTTCACCTCCT-3′) and Peg3-KO-64 (5′-CACTGCCAAAAGCATGAGATGGTC-3′). The sex of each MEF was also determined using the following primer set: mSry-F (5′-GTCCCGTGGTGAGAGGCACAAG-3′) and mSry-R (5′-GCAGCTCTACTCCAGTCTTGCC-3′).

### Chromatin ImmunoPrecipitation (ChIP)

Chromatins were prepared from MEF according to the method previously described^5^. In brief, the homogenized samples were first cross-linked with 1% formaldehyde for 20 mins, and then lysed with the buffer containing protease inhibitor cocktail (Millipore, Cat. No. 539131). The released nuclei were fractionated with sonication to derive a pool of DNA fragments size-ranging from 300 to 500 bp in length. The prepared chromatin was immunoprecipitated with two commercial antibodies: anti-YY1 antibody (SantaCruz Biotech, Cat. No. sc-1703) and anti-H3K27ac antibody (Abcam, Cat. No. ab4729). The immunoprecipitated DNA was dissolved in 80 μl of TE for PCR analyses.

## Electronic supplementary material

Supplementary Information
