# Supplementary material for: YY1’s role in the Peg3 imprinted domain
Source: Sci Rep. 2017 Jul 25;7:6427. doi: 10.1038/s41598-017-06817-5 (PMC5526879; doi:10.1038/s41598-017-06817-5)

YY1’s roles in the Peg3 imprinted domain

Hongzhi He, An Ye, Bambarendage P.U. Perera, Joomyeong Kim

Department of Biological sciences, Louisiana State University,
Baton Rouge, LA 70803, USA

Hhe_Supplemental_material_1

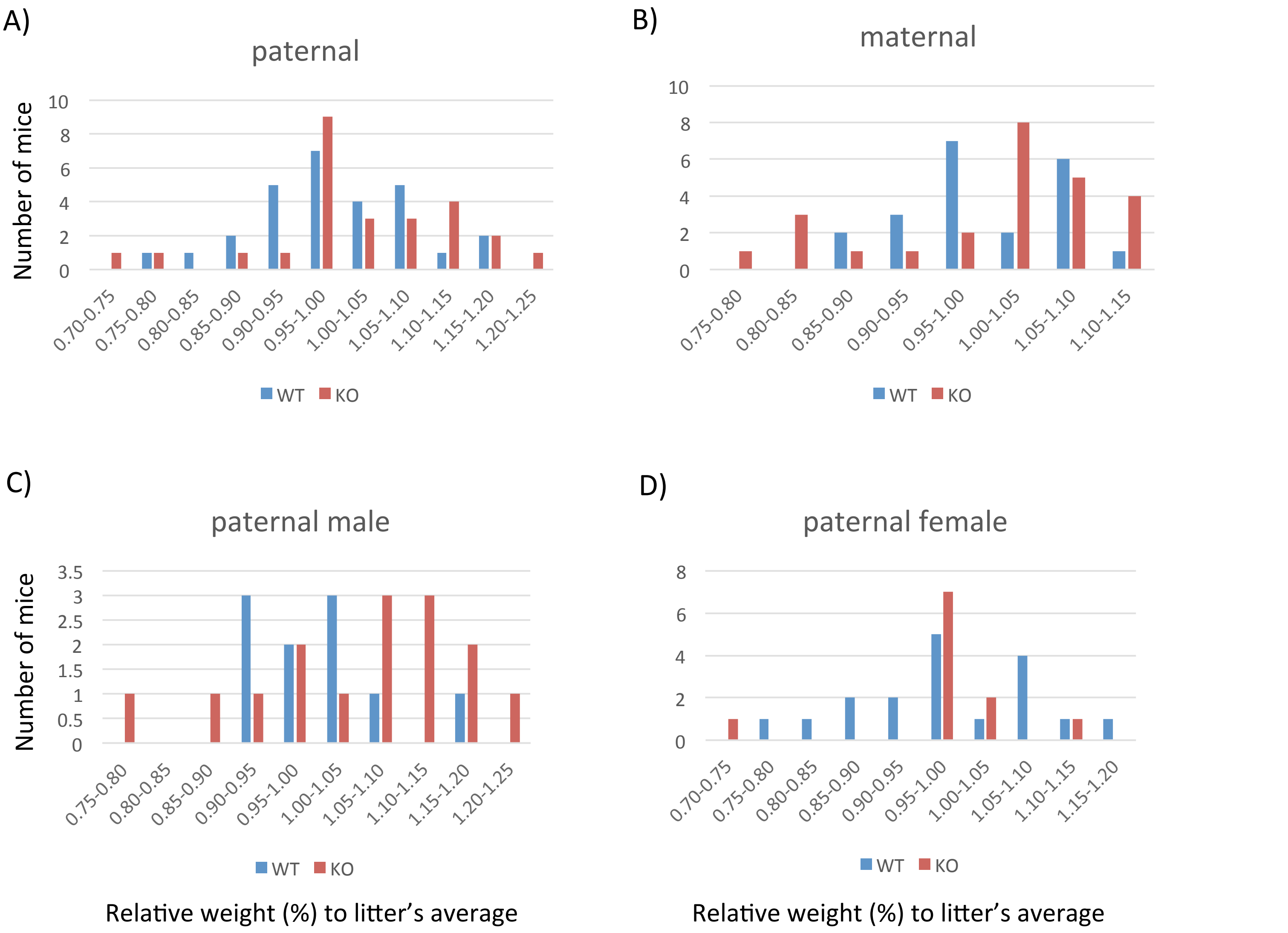

Supplemental material 1. Male and female heterozygotes were individually bred with their wild-type littermates for the paternal and maternal transmission of the mutant allele (**A**,**B**). The weight profiles of the pups with the paternal transmission of the mutant allele were divided based on their sexes, and subsequently re-analyzed (**C**,**D**).

Hhe_Supplemental_material_2

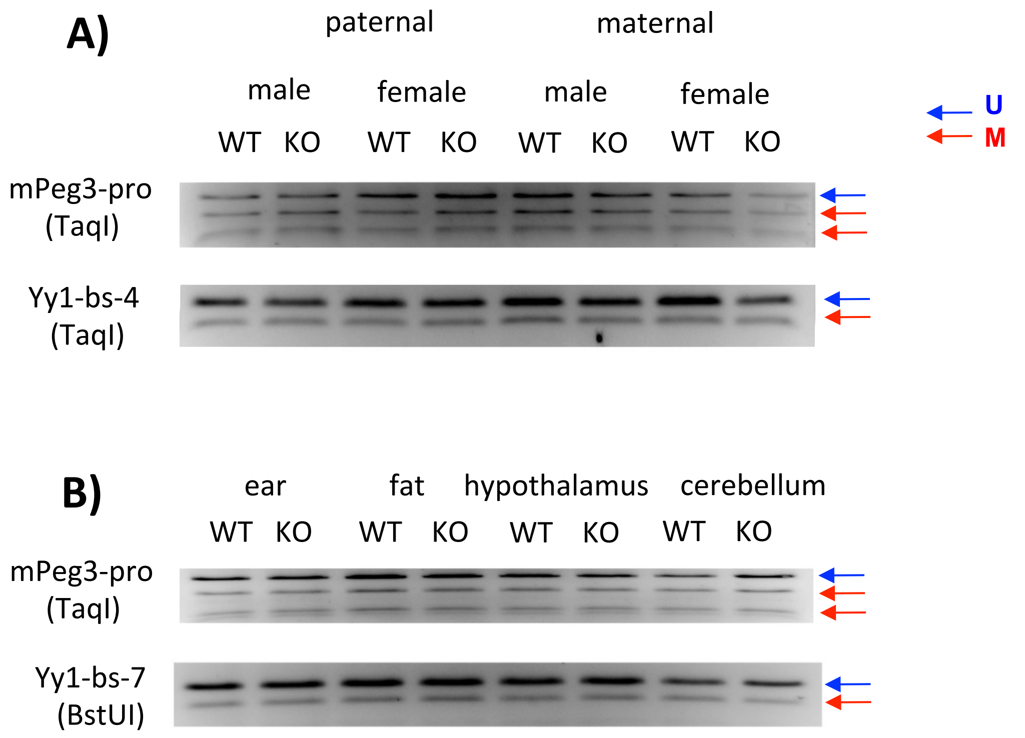

Supplemental material 2. DNA methylation analyses using the DNA isolated from the neonatal brains of wild type (WT) and knockout (KO) mice with the paternal and maternal transmission of the mutant allele (**A**). DNA methylation analyses using the tissues of adult mice with the maternal transmission of the mutant allele (**B**).

Hhe_Supplemental_material_3

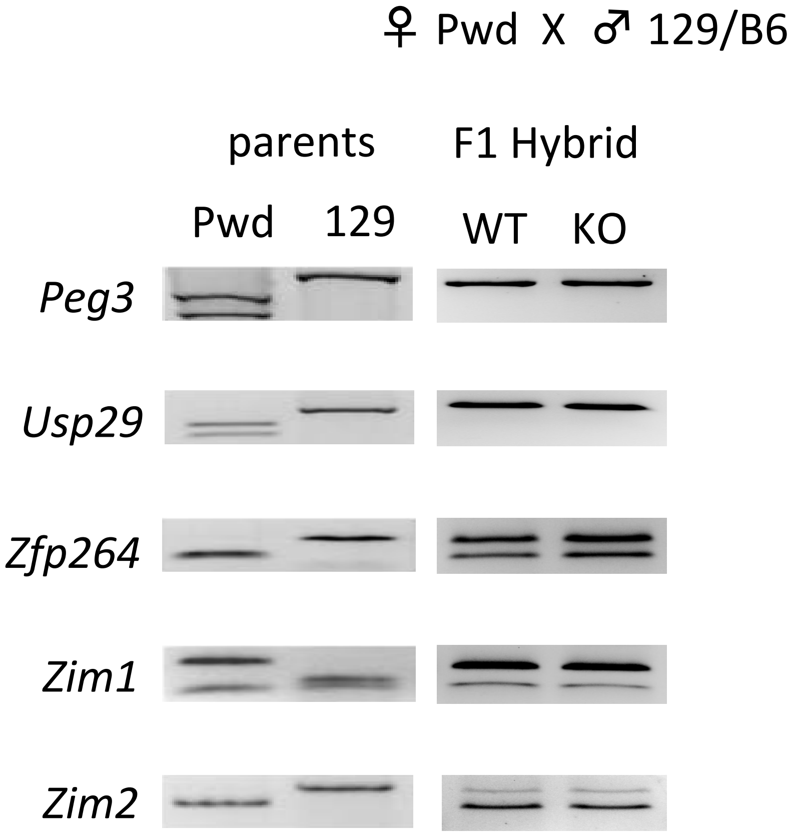

Supplemental material 3. The one-day-old heads of F1 hybrids from the crossing between the male KO of 129/B6 and the female breeders of Pwd were used for imprinting test. The digestion patterns for the 5 tested genes from the two parental alleles are shown on the two left columns, while the two right columns are the results from the F1 hybrids. The results indicated no major changes in the imprinting status of the 5 imprinted genes between WT and KO with the paternal transmission of the mutant allele.

Hhe_Supplemental_material_4

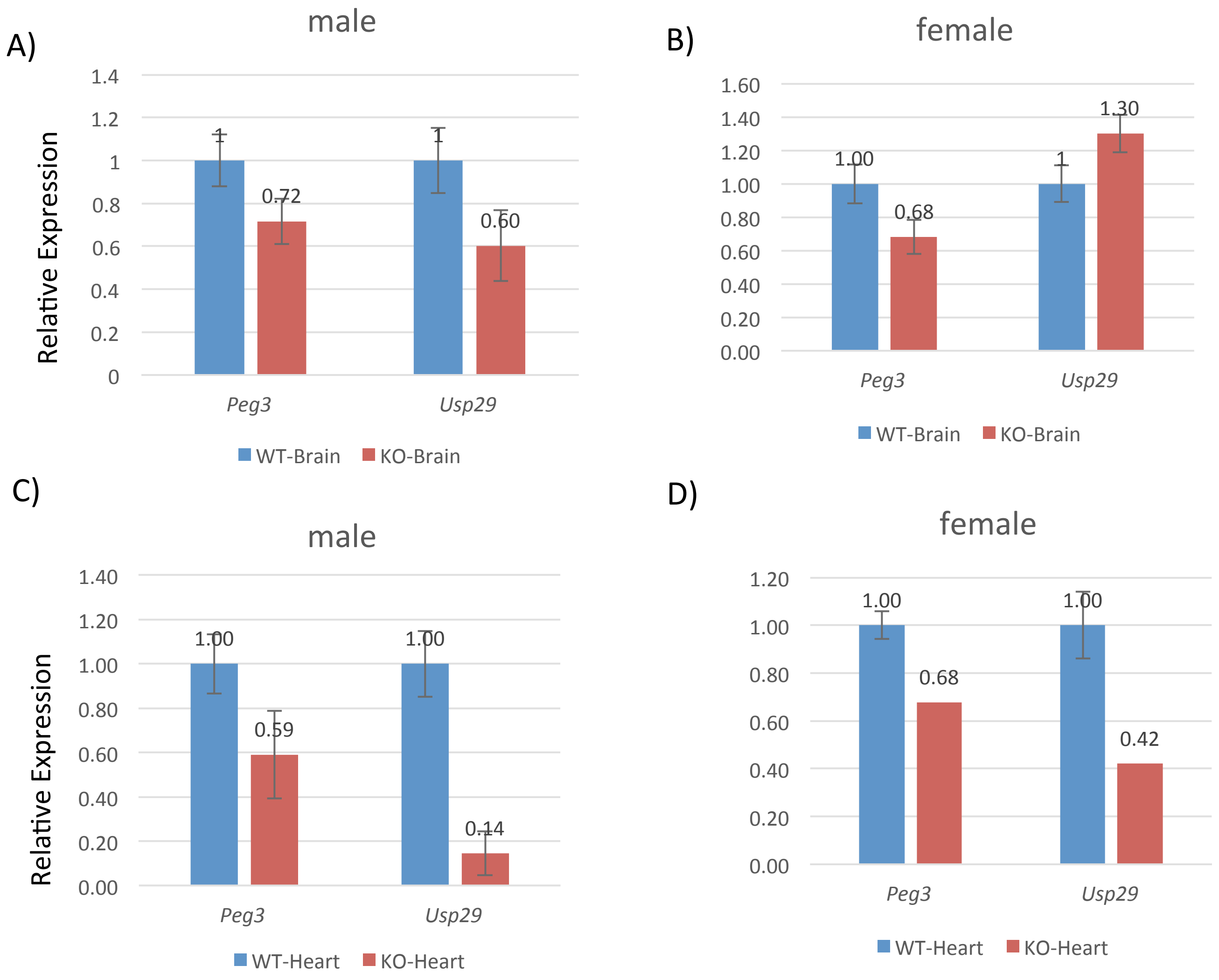

Supplemental material 4. Mutational effects on the expression levels of *Peg3* and *Usp29* in the brains and hearts of the 2-month-old mice with the paternal transmission of the mutant allele. The series of analyses were repeated with two independent sets of biological replicates.

Supplementary Material 5. Primer sets used for imprinting test.

Locus	Name	Sequence (5' -> 3')	Primer set	Size (bp)	Genbank No:Position	RFLP enzyme (cut vs uncut)
Peg3	Peg3-RTl-1a<br>Peg3-RT-1b.3	GGTTCAGTGTGGGTGCACTAGACT<br>TCCCTAGTGTGCATGATCTGGT	1st primer	1319	NM_008817:93-1411	BstUI (PWD vs 129)
Usp29	Usp29-RT-Pol-a<br>Usp29-RT-Pol-b.1	GAGCCTGCAGCCGGACCGT<br>GCTGCGGATCTGGACCAAACCA	1st primer	329	NM_021323:972-1300	Tsp45I (PWD vs 129)
Zim1	Zim1-F1<br>Zim1-F2	GAATTCACACGGGAGTGAGA<br>CTTGCACCGGTACCTGGAGT	1st primer	539	NM_011769:1357-1895	DraI (PWD vs 129)
Zim2	Zim2-17<br>Zim2-19	GCTCAGGACCCTGCGCTTTCAG<br>GGGCTGCCAAGATCAATGCTG	1st primer	538	AF401983:57-594	BstUI (PWD vs 129)
Zim3	Zim3-RT-Pol-a<br>Zim3-RT-Pol-b.1	CCCTTACTGACCTAGGGCTTG<br>AGTGACGTCCTCTAAGGTCACT	1st primer	269	NR_036631.2<br>:101-369	EcoRV (129 vs PWD)
Zfp264	Zfp264-RT-Pol-b.1<br>Zfp264-RT-Pol-b	CTGGGCATGAAAAGATCCACT<br>CCACATTTGTTGCACTGGTGGAT	1st primer	229	AF365933:404-632	BstUI (PWD vs 129)
Apeg3	APeg3-R<br>APeg3-F	GCACCAGTGCAGGTGGTGCGGA<br>CAATCAGTCTCAAGGGGTCTGGGT	1st primer	865	NR_023846:1-865	XhoI (PWD vs 129)

Supplementary Table 2. Primer sets used for the bisulfite sequencing and COBRA.

Locus	Name	Sequence (5' -> 3')	Primer set	Size (bp)	*Position (mm10, NCBI Build 38)
Peg3-pro	Peg3-pro-a<br>Peg3-pro-b	AATAGTAGTTTGATTGGTAGGGTGTGGGA<br>CACCCCAAACACCATCTAAACTCTACAAAC	1st primer	380	chr7: 6730175-6730555
	Peg3-pro-a.1<br>Peg3-pro-b	GTTTTTGTAGAGGATTTTGATAAGGAG<br>CACCCCAAACACCATCTAAACTCTACAAAC	2nd primer (Nested)	292	chr7: 6730175-6730467
Yy1-bs-2	Yy1-15<br>Yy1-16.1	AGGAAGAGTTAGAGGAGTTAGTATTTTATAGA<br>CCTATTACAAAACCACCACAATAAACATCA	1st primer	509	chr7: 6729374-6729883
	Yy1-15.2<br>Yy1-16.bis2 (for KO)<br>Yy1-16.1 (for WT)	TAGGTAGTTAATTAGGATAAGTTTGTGTAG<br>TTCCTATACTTTCTAAAAAATAAAAACTTC<br>CCTATTACAAAACCACCACAATAAACATCA	2nd primer (Nested)	345	chr7: 6729538-6683362
Yy1-bs-4	Yy1-19<br>Yy1-22	TTTTATGGGTTTGTGAAGTTTTTAG<br>CAAATCCCACCCCCAACCAACCTATC	1st primer	216	chr7: 6728371-6728587
	Yy1-19<br>Yy1-20	TTTTATGGGTTTGTGAAGTTTTTAG<br>CCAACCAACCTATCATTCAAAATA	2nd primer (Nested)	204	chr7: 6728383-6728587
Yy1-bs-7	Yy1-25<br>Yy1-26	TTTTTTTTGAGTGATTGGGTTATAGAAGTT<br>ATAACACATCCACAATAAAAAATTCAAACA	1st primer	358	chr7: 6727253-6727611
	Yy1-25<br>Yy1-26.bis (for KO)<br>Yy1-26.1 (for WT)	TTTTTTTTGAGTGATTGGGTTATAGAAGTT<br>CAATAAAAAATTCAAACACCAAATCCATAACTTC<br>CAATAAAAAATTCAAACACCCCCTTATAAC	2nd primer (Nested)	346	chr7: 6727265-6727611
H19-ICR	H19-ICR-BF3<br>H19-ICR-BR3-nes	ATAGATGGTGATAGGGGAGAAAATTTA<br>AAATTCTACAAAAAAACCATACCCTATTCTT	1st primer	402	chr7: 142581719-142582121
	H19-ICR-BF3<br>H19-ICR-BR3-nes	AGATGGTGATAGGGGAGAAAATTTAATTAGTTGT<br>ATTCTACAAAAAAACCATACCCTATTCTTAAAC	2nd primer (Nested)	398	chr7: 142581721-142582119

Supplementary Table 3. Primer sets used for qRT-PCR experiments.

Locus	Name	Sequence (5' -> 3')	Primer set	Size (bp)	*Position (mm9, NCBI Build 37)
Peg3	Peg3-RT-1a<br>Peg3-RT-1b	GGTTCAGTGTGGGTGCACTAGACT<br>GCTCACACCCAAGGGCTTGAGCG	1st primer	222	chr7:6,671,201-6,683,038
Usp29	Usp29-RT-1a<br>Usp29-RT-1c	GAGGAGAGCAAGCAGGTAGATTAC<br>GTTGAAATGGGGAGTAGGGTGA	1st primer	545	chr7:6683856-6690299
Zim1	Zim1-RT-c<br>Zim1-RT-d	GATCACCAGGTTGGAGCAAGGAGT<br>AGCGCTCTGTGGTGTTGTAGTTG	1st primer	305	chr7:6630678-6634867
Zim2	Zim2-17<br>Zim2-RT-19	GCTCAGGACCCTGCGCTTTCAG<br>GGGCTGCCAAGATCAATGCTG	1st primer	211	chr7:6605023-6611658
Zim3	Zim3-RT-a<br>Zim3-RT-b	GCCAGGCATAGCGGTACTTGCCT<br>CAGTCATCTCTGTCACTCACAGA	1st primer	438	chr7:6925908-6929182
Zfp264	Zfp264-RT-Pol-b<br>Zfp264-RT-Pol-b.1	CTGGGCATGAAAAGATCCACT<br>CCACATTTGTTGCACTGGTGGAT	1st primer	337	chr7:6941897-6942125
Actin (beta)	bActin-1a<br>bActin-1b	GAGCACCCTGTGCTGCTCACCGA<br>CTCTTTGATGTCACGCACGATTTC	1st primer	345	chr5:143,666,183-143,666,981

Supplementary Table 4. Primer sets used for CHIP Assay

Locus	Sequence (5' -> 3')	Sequence (5' -> 3')	Primer set	Size (bp)	*Position (mm9, NCBI Build 37)
Peg3-DMR	YY1-01a<br>YY1-01b	GTCCTGTTACAAGACCACCAC<br>CTGGGTGGAGCCGAAAAGTATC	1st primer	284	chr7:6682313-6682596
Peg3-DMR	YY1-23a<br>YY1-23b	AAAGATGCACCCCAGTTGTC<br>CGGGCGACACTTTTTAACTC	1st primer	270	chr7:6681801-6682070
Peg3-DMR	YY1-34a<br>YY1-34b	CTACCCTGACGCCATCTTTG<br>GATGACACCCGCTGAGAACT	1st primer	205	chr7:6681263-6681467
Peg3-DMR	YY1-45a<br>YY1-45b	TCACTGAAGGTGCCATTTTG<br>CAGAGCTCCCTGCTCATTCT	1st primer	300	chr7:6680693-6680992
Peg3-DMR	YY1-56a<br>YY1-56b	CACTAGAGGCAGGCAAGAGG<br>ACTGGCGGAATGAATACCAG	1st primer	250	chr7:6680232-6680481

Hhe_Supplemental_material_6

Original images for Fig.3A

Sperm
H19 ClaI & mPeg3-pro FokI

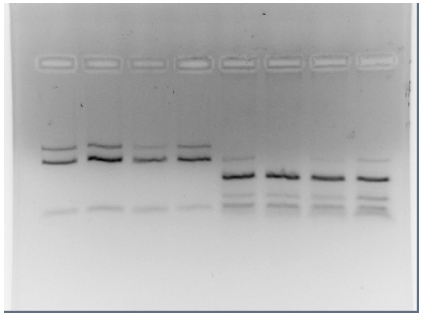

Sperm (6month)
Yy1-bs-7 (TaqI)

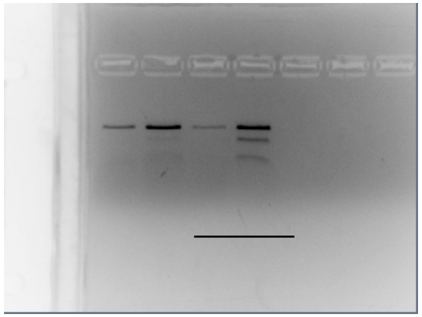

Somatic DNA
H19, Peg3-pro, Yy1-bs-2

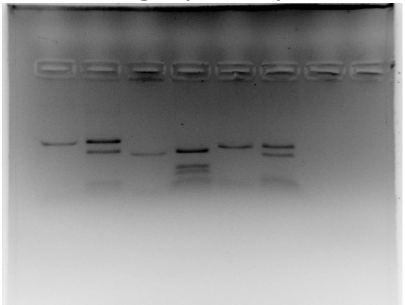

Sperm
Yy1-bs-2 & Yy1-bs-4

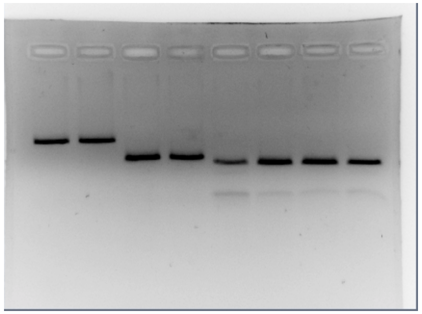

Egg
Yy1-bs-2 & Yy1-bs-4

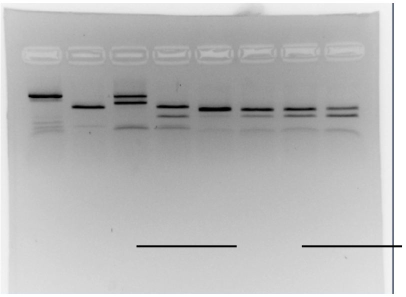

Somatic DNA Sperm
Yy1-bs-4, 7 & Peg3-pro TaqI

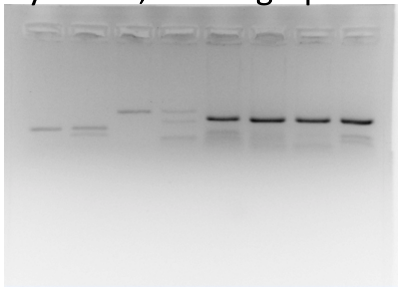

Sperm
Yy1-bs-7

Egg
H19 & mPeg3-pro

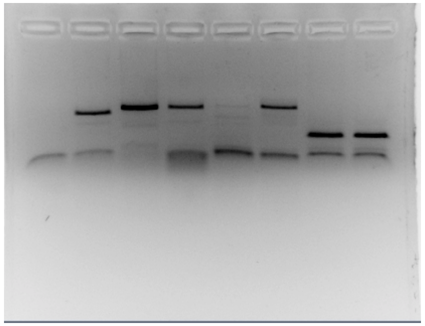

Egg
Yy1-bs-4 (BstUI)- middle set

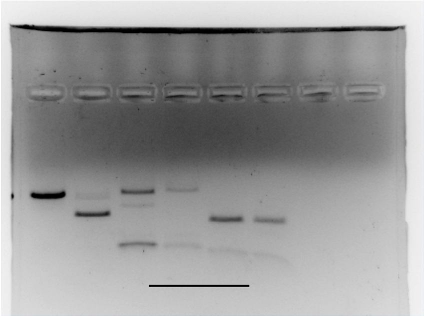

Sperm
Yy1-bs-7 new one

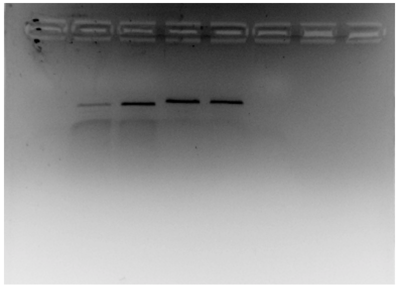

Original images for Fig.5C

MEFs COBRA
mPeg3-pro & Yy1-bis-7

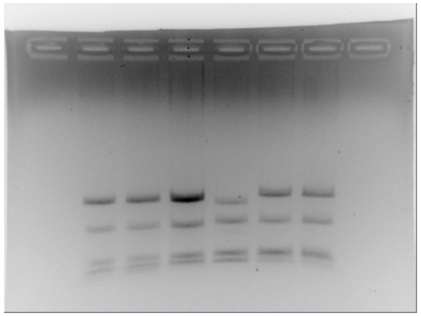

MEFs COBRA
Yy1-bs-2 & Yy1-bs-4

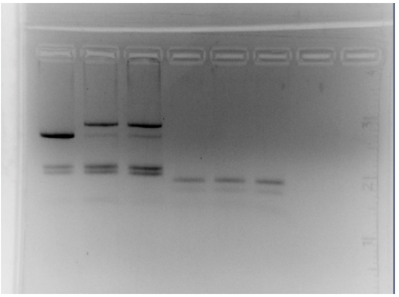

MEFs COBRA
mZim2 & Zfp264

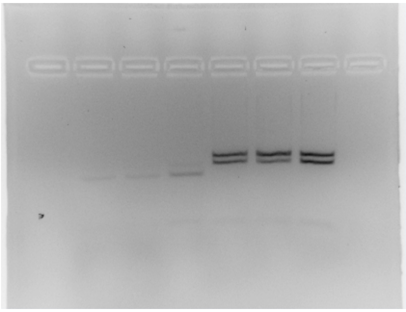

Hhe_Supplemental_material_7

Control PCR reactions for bisulfite-converted DNA

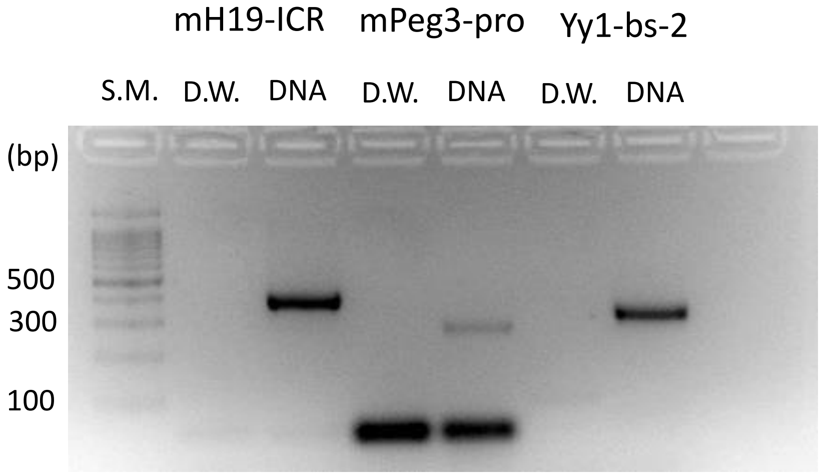

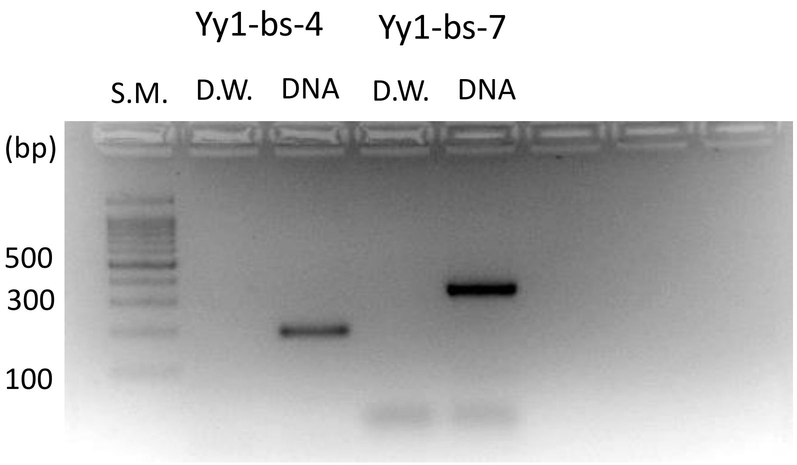

Control COBRA experiments

S.M.: Size Marker

D.W: Distilled Water

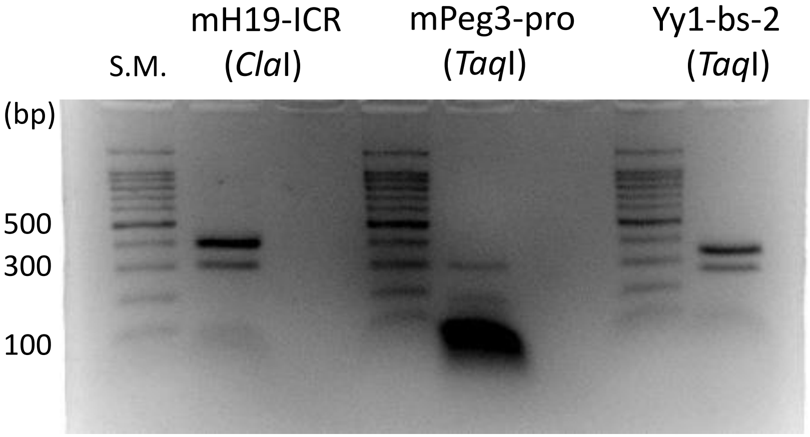

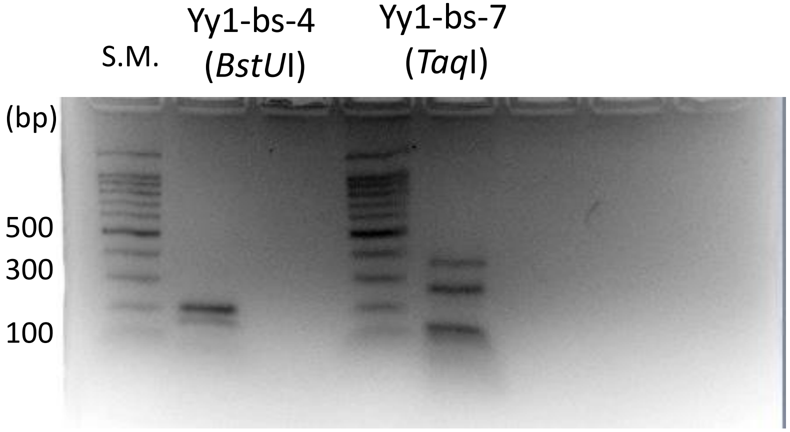

Supplement: Supplementary file 1 — Supplementary Information [file 41598_2017_6817_MOESM1_ESM.pdf]
